# Nitrogen supply mitigates heat-driven potato yield loss by sustaining radiation use efficiency and dry matter partitioning to tubers

**DOI:** 10.3389/fpls.2026.1846993

**Published:** 2026-06-10

**Authors:** Yiyin He, Chao Li, Jiayi Si, Liang Zhang, Zhenjiang Zhou

**Affiliations:** 1 College of Biosystems Engineering and Food Science, Zhejiang University, Hangzhou, China; 2 Key Laboratory of Spectroscopy Sensing, Ministry of Agriculture and Rural Affairs, Hangzhou, China; 3 Key Laboratory for Innovative Utilization of Characteristic Food Crop Resources in Central Zhejiang, Jinhua, China

**Keywords:** dry matter partitioning, heat stress, nitrogen fertilization, potato, radiation use efficiency, tuber yield formation

## Abstract

Heat episodes increasingly constrain potato (*Solanum tuberosum* L.) productivity, yet how nitrogen (N) status modulates heat-induced limitations in canopy function, radiation use efficiency (*RUE*), and source–sink allocation remains insufficiently resolved. We conducted a pot experiment in controlled climate chambers using two temperature regimes (control, T0; heat stress, T1) combined with three N rates (0, 50 and 150 kg ha^-^¹; N0, N1 and N2) in a split-plot design. Compared with T0, heat stress significantly raised canopy temperature, reduced chlorophyll status (*SPAD*), and decreased *RUE* by 15.4%, and restricted the translocation of photoassimilates from shoots to tubers, which resulted in a 42.1% decline in tuber dry weight at harvest. Crucially, while heat stress drove a 31.9% reduction in *RUE* under N-deficient conditions (N0), this penalty was effectively neutralized under high N (N2). Furthermore, sufficient N supply alleviated the heat-induced restriction on dry-matter translocation, reducing the biomass fraction retained in shoots from a 6.9% excess at N0 to only 3.9% at N2, thereby facilitating more effective dry matter allocation to tubers under thermal stress. Consequently, heat-induced tuber yield loss was alleviated by N, with dry weight reductions declining from 55% (N0) to 33% (N2). Overall, sufficient N supply mitigated heat-induced yield loss in potato primarily by sustaining *RUE* and dry matter partitioning to tubers during bulking, providing physiological insight into how N status supports tuber yield formation under heat stress.

## 1 Introduction

Global warming is increasingly exposing potato crops to episodes of supra-optimal temperature, resulting in substantial reductions in tuber yield and dry matter accumulation (Hijmans, 2003; Hancock et al., 2014; Dahal et al., 2019). Potato (*Solanum tuberosum* L.) exhibits optimal vegetative growth and tuber development at moderate temperatures, typically ranging from 15 to 25 °C (Levy and Veilleux, 2007; Rykaczewska, 2013). Consequently, potato is highly vulnerable to supra-optimal temperatures, with physiological functions and tuberization being severely restricted when ambient temperatures exceed 25 to 30 °C (Levy and Veilleux, 2007; Rykaczewska, 2013; Thornton et al., 2014; Dahal et al., 2019).

Radiation use efficiency (*RUE*), defined as the efficiency with which intercepted radiation is converted into biomass, provides an effective integrative indicator linking canopy photosynthesis with crop growth (Monteith, 1977; Sinclair and Muchow, 1999). Previous studies have demonstrated that heat stress can substantially reduce *RUE* in potato and other crops by impairing canopy photosynthetic performance and shortening canopy duration (Fleisher et al., 2006; Parent and Tardieu, 2012). Heat stress can also accelerate chlorophyll degradation and impair photosynthetic processes, resulting in reduced canopy functional performance and lower biomass production (Mathur et al., 2014; Gururani et al., 2015). However, yield reduction under heat stress cannot be explained by *RUE* decline alone. Elevated temperature delays stolon development, suppresses tuberization, and shortens the effective period for tuber growth (Struik et al., 1989; Levy and Veilleux, 2007; Zhou et al., 2017; Singh et al., 2020; Zhang et al., 2024). During tuber bulking, high temperature reduces the allocation of photoassimilates to tubers and limits their conversion into starch, thereby weakening sink development and final dry matter accumulation (Wolf et al., 1991; Paul et al., 2017; Lehretz et al., 2020). Increasing evidence suggests that both biomass production efficiency and assimilate partitioning jointly determine potato heat resilience, yet their relative contributions remain insufficiently quantified.

Nitrogen (N) supply is a major regulator of canopy productivity and carbon allocation in potato. Adequate N supply promotes canopy expansion, maintains chlorophyll content and photosynthetic activity, and delays premature senescence, thereby supporting biomass accumulation and *RUE* maintenance (Millard and Marshall, 1986; Waraich et al., 2012; Bangemann et al., 2014; Artins et al., 2024). Indicators such as canopy spectral traits, canopy area, *SPAD*, and tissue N concentration have also been widely used to characterize potato N status (Jongschaap and Booij, 2004; Saravia et al., 2016; Peng et al., 2021). In addition to sustaining canopy photosynthetic activity, N supply also influences assimilate partitioning and tuber sink development in potato (Ding et al., 2024). N fertilization has been reported to alleviate heat-induced yield loss in some crops, although the extent and direction of the response can vary depending on species and stress context (Waraich et al., 2012; Liu et al., 2013, 2019). Previous mechanistic discussions on N-mediated heat tolerance in potato have mainly focused on general physiological or molecular phenomena, while the precise mechanisms by which varying N supply regulates *RUE* and dry matter allocation under heat stress remain poorly understood.

Specifically, previous studies have rarely quantified whether N mitigates heat-induced yield loss primarily through sustaining canopy biomass production, improving assimilate allocation to tubers, or through coordinated regulation during tuber bulking. We hypothesized that adequate N alleviates heat-induced yield loss by synergistically sustaining canopy *RUE* and mitigating the heat-induced restriction of photoassimilate translocation from shoots to tubers during bulking. Therefore, this study investigated the main and interactive effects of temperature and N supply on canopy traits, plant N status, biomass accumulation and partitioning, and tuber yield formation under controlled conditions using contrasting temperature regimes and three N application rates. Combined with the accumulated intercepted photosynthetic active radiation (*Aipar*) × *RUE* framework, this study is aimed to quantify the relative contributions of these two mechanisms to N-mediated heat resilience in potato, addressing the critical gap in systematic analysis of their coordinated regulation under heat stress.

## 2 Materials and methods

### 2.1 Experimental design

A pot experiment was conducted in controlled-climate chambers in Hangzhou, Zhejiang Province, China, in 2022. The potato cultivar ‘*Zhongshu 5’*, a widely cultivated and early-maturing commercial variety in China, was used for the experiment. This variety exhibits broad adaptability across diverse agro-ecological zones and is typically grown during relatively cool periods of the year, making it a representative model for studying heat stress responses in mainstream commercial production systems. The specific seed potatoes used in this trial were virus-free minitubers provided by the Institute of Vegetables and Flowers, Chinese Academy of Agricultural Sciences. Prior to sowing, the tubers were pre-sprouted at room temperature (approximately 18 °C) for about 30 days. To ensure the uniformity of initial plant emergence and early growth, evenly sprouted tubers weighing between 40 g and 60 g were strictly selected.

A single sprouted tuber was planted in each polyvinyl chloride pot. Each polyvinyl chloride pot had a top diameter of 27.5 cm, a bottom diameter of 23.5 cm, and a height of 24.0 cm. The experimental soil comprised a mixture of field soil and commercial substrate weighing 8.5 kg and 0.8 kg per pot, respectively. The field soil is characterized as loamy with total N content of 1.30 g kg*^-^*^1^, organic matter of 24.1 g kg*^-^*^1^, and hydrolytic N content of 100 mg kg*^-^*^1^. The commercial substrate contains total N content of 8.73 g kg^-1^ and hydrolytic N content of 397 mg kg*^-^*^1^. To effectively isolate nitrogen as the sole nutritional variable across the treatments, a highly sufficient amount of P and K was uniformly applied to all pots to ensure these macronutrients were not limiting factors. Specifically, 5.7g of potassium dihydrogen phosphate (KH_2_PO_4_) at sowing, and 11g of potassium sulfate (K_2_SO_4_) during the growth period were added per pot. The tubers were sown on 13^th^ June, and the final harvest was conducted on 4^th^ Oct.

**Figure 1:**
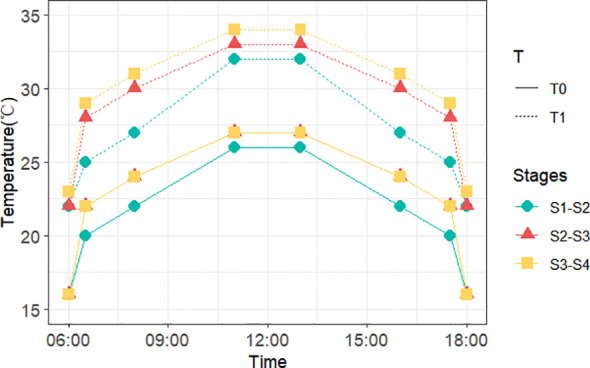
Daily temperature settings for T0 and T1 during different growth stages of S1-S2, S2-S3, S3-S4. S1, S2, S3 and S4 represent the data collection date (S1: 13^th^ Aug, S2: 25^th^ Aug, S3: 17^th^ Sept, S4: 4^th^ Oct).

The experiment was arranged in a split-plot design with 12 replications per treatment. A total of six treatments were applied differentiated by varying N rates (0, 50, and 150 kg ha^-1^) defined as N0, N1, and N2, as well as two temperature regimes (control and heat stress), defined as T0 and T1. The selected N application rates (0, 50, and 150 kg ha^-1^) were chosen to represent N-deficient, moderate, and adequate N supply conditions, respectively, based on previous potato fertilization studies and regional recommendations for potato production, which indicate that approximately 150 kg ha^-^¹ serves as an optimal N supply threshold to maximize potato tuber yield (Wang et al., 2020; Liu et al., 2021; Fang et al., 2023). N fertilizer was applied at sowing using urea, with 0, 2.1g, and 7.5g each pot for N0, N1, and N2, respectively. Two climate chambers were used to simulate different diurnal temperature conditions, as shown in **Figure 1**. The diurnal temperature profiles for T0 were set to mimic the ambient temperatures in Zhangjiakou, Hebei Province (a typical potato-growing region in China) during the corresponding growth stages. To induce significant heat stress, the temperatures in T1 were maintained 5–7 °C higher than those in T0 throughout the experiment, successfully shifting the daily temperatures beyond the optimal range (15–25 °C) for potato growth, which is known to induce significant heat stress and tuber yield reduction (Levy and Veilleux, 2007; Rykaczewska, 2013). Relative humidity was kept at 40%. The climate chamber was illuminated with LED plant growth lamps (LQ-LED/plant-150-M1 and LQ-LED/plant-150-M2; Hangzhou Liquan Technology Co., Ltd., Hangzhou, China), providing an average photosynthetic photon flux density (PPFD) of approximately 505 μmol m^-^² s^-^¹ at canopy level, based on measurements taken at 10 positions within the chamber, under a 12h photoperiod. Irrigation was carried out based on maintaining soil moisture at 60-80% of the field capacity, as monitored using an HD2 soil moisture probe (IMKO Micromodultechnik GmbH, Germany).

### 2.2 Sample collection and data acquisition

Data collection was conducted once at each growth stage: early tuberization stage (S1), late tuberization stage (S2), tuber expansion stage (S3), and late tuber expansion stage (S4). These corresponded to 13^th^ Aug, 25^th^ Aug, 17^th^ Sept and 4^th^ Oct. Three replications from each treatment were measured and sampled for data collection. For each sampling date, 18 samples were collected, resulting in a total of 72 samples over the entire growth period. Destructive sampling was conducted for each sampling dates, while non-destructive data collections including canopy RGB image, thermal image, tuber image and *SPAD* were only conducted on 13^th^ Aug, 25^th^ Aug and 17^th^ Sept.

#### 2.2.1 Non-destructive measurements

To determine canopy area and canopy coverage, top-view canopy digital images of individual plants were acquired using a smartphone camera positioned at an approximate height of 1.2 m above the ground. Images were recorded at a resolution of 4000 × 2256 pixels and saved in JPEG format. To facilitate canopy segmentation, a black cloth was placed beneath each pot to provide a uniform background. At the same time, a scale card of known area was positioned beside the canopy in each image for area calibration. To calculate canopy temperature, canopy thermal image of single potato plant was obtained using a hand-held infrared imaging device (Fluke Ti401 PRO, Inc., USA) with an infrared resolution of 640 × 480, with IS2 file storage format, and ± 2% measurement accuracy. The thermal camera had a 34°H × 24°V field of view, and was kept at an approximate 1.0 m above the canopy. The thermal images were collected from 10:00-10:30 am. For each selected plant, three leaves from the upper, middle, and lower parts of the plant were selected to measure *SPAD* values with *SPAD*-502 (Konica Minolta Inc., Tokyo, Japan), which was averaged for each plant and employed as indication of leaf chlorophyll content.

#### 2.2.2 Destructive sampling and chemical analysis

Destructive sampling was carried out shortly after non-destructive measurements, each potato plant was cut and divided into shoot, root, and tubers. Each single tuber was weighed with a balance to obtain its fresh weight. This individual measurement was conducted to evaluate the tuber size distribution, enabling the differentiation between yield penalties caused by impaired tuber initiation (tuber number) versus impaired tuber filling (individual tuber weight). Subsequently, all samples were dried in an oven at 105 °C for 30 minutes, followed by drying at 75 °C for 48 hours, to determine the dry matter (*DM*) of shoot, root and tuber. Afterwards, the dried shoot and tuber tissues were finely ground and weighed into tin capsules using an ML204 analytical balance (Mettler Toledo International Inc., Greifensee, Zurich, Switzerland) for elemental analysis. The carbon (C) and N concentrations were then quantified using an elemental analyzer (Elementar EL III, Hanau, Germany). The N accumulation (*N*_acc_, g) was calculated using Equation 1:

(1) $$N_{acc}=N_{con}\times DM$$

where *N*_con_ is the N concentration (g/kg) and *DM* is the corresponding organ dry weight (kg). Subsequently, *C/N* was calculated using Equation 2:

(2) $$C/N=\frac{C_{\text{acc}}\;}{N_{\text{acc}}}$$

where *C*_acc_ represents the C accumulation (g) calculated in the same manner as N accumulation.

### 2.3 Canopy RGB image processing and canopy area estimation

Canopy RGB images obtained as described in Section 2.2.1 were processed using Python 3.8 (Python Software Foundation, Vienna, VA, USA). Each image was separated into red (*R*), green (*G*), and blue (*B*) channels, and the excess green index (*ExG*) was calculated using Equation 3:

(3) ExG = 2 × G−R−B

**Figure 2:**
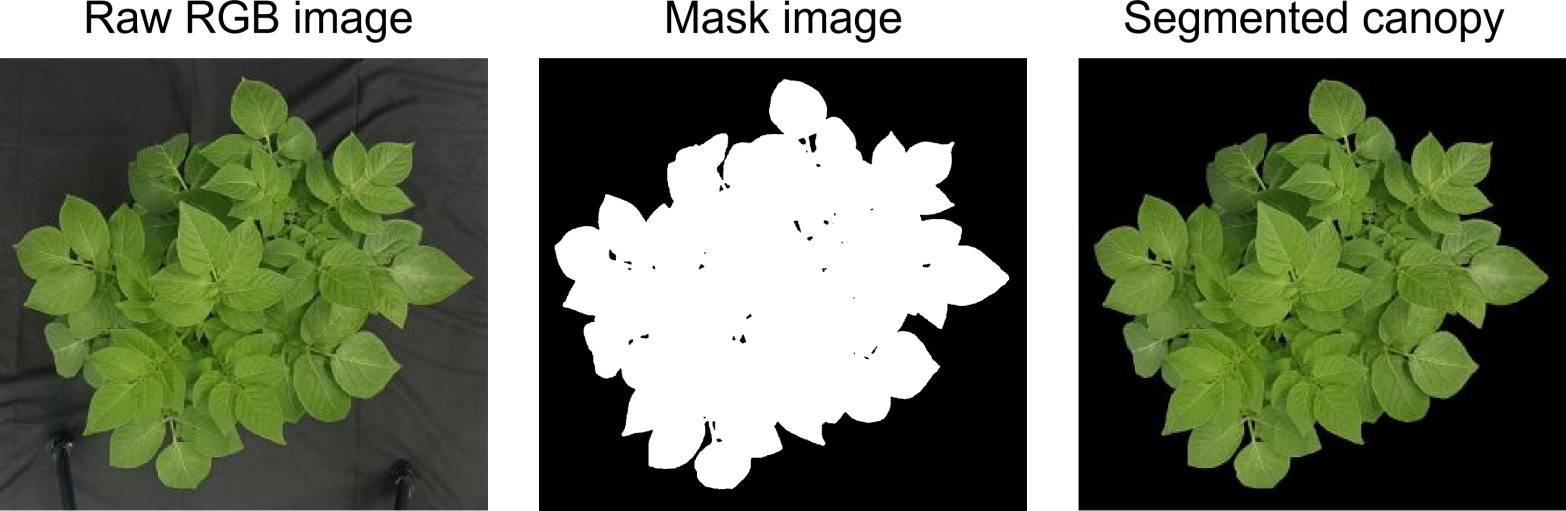
The raw RGB image, mask image, and segmented canopy image in RGB image processing.

Pixels with *ExG* values greater than 0.08 were assigned a value of 1 and classified as green vegetation, whereas all other pixels were assigned a value of 0 and classified as background (**Figure 2**).

Based on the resulting binary images, the observed canopy area ( $A_{obs}$) was obtained by converting canopy pixels to area using the scale card included in each image. Because plant height increased during growth, the apparent canopy area in the images was corrected for the change in camera-to-canopy distance. The corrected canopy area ( $A_{corr}$) was calculated as follows (Equation 4):

(4) $$A_{corr}=A_{obs}\;\times {\left(\frac{H-(h_{plant}+h_{pot})}{H}\right)}^{2}$$

where $A_{corr}$ is the corrected canopy area, $A_{obs}$ is the observed canopy area derived from image segmentation, H is the fixed vertical distance between the camera and the reference ground plane, $h_{plant}$​ is plant height, and $h_{pot}$ is pot height. The corrected canopy area was used to describe canopy development in the subsequent analysis.

For radiation interception analysis, canopy coverage (*CC*) was calculated as the ratio of corrected canopy area to the fixed reference ground area ( $A_{ref}$) (Equation 5):

(5) $$CC=\;\frac{A_{corr}}{A_{ref}}$$

where *CC* is canopy coverage, and $A_{ref}$ is the fixed ground reference area corresponding to the image field of view. In this study, $A_{ref}\;$was determined as an idealized ground reference area of 1 m^2^. Thus, canopy area was used as a morphological indicator of canopy development, whereas canopy coverage was used as a proportional variable for the subsequent estimation of intercepted radiation.

### 2.4 Canopy thermal image processing and canopy temperature statistics

**Figure 3:**
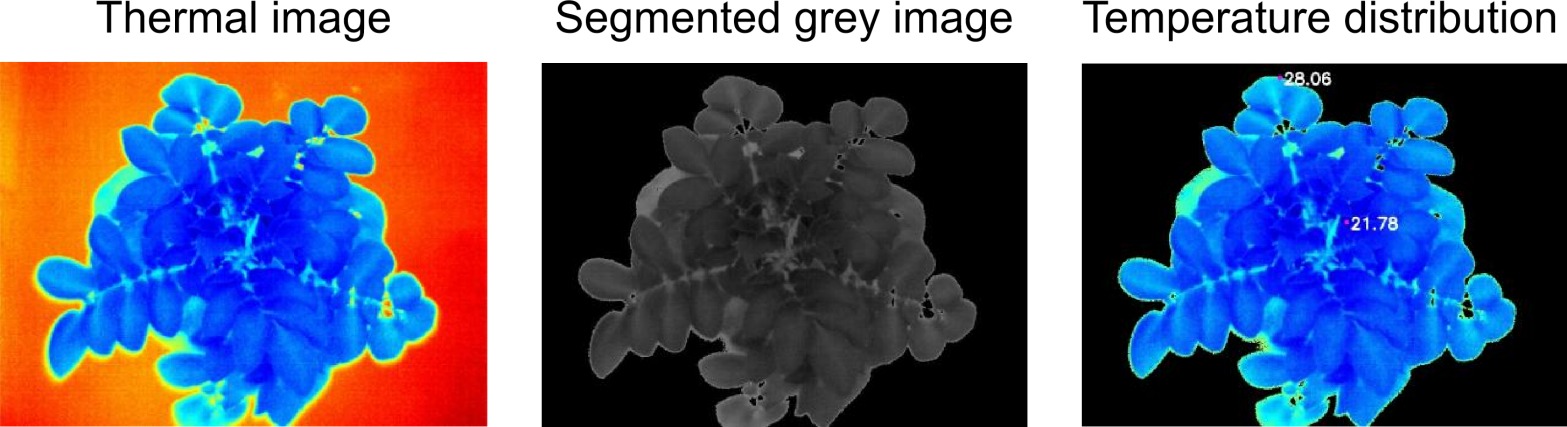
Thermal image processing workflow.

The temperature data in XLS format could be generated in batches from the supporting software (Fluke Connect, Inc., USA). Then, Matlab (The MathWorks, Inc., Natick, MA, USA) programming was used to normalize the temperature matrix from the XLS file for each sample. Subsequently, adaptive threshold segmentation was conducted to delineate the canopy region and extract the temperature data pertaining to this area, with a statistical analysis to determine the maximum value, minimum value, and overall temperature distribution of every treatment (**Figure 3**).

### 2.5 Radiation interception measurement by light quantum sensor

Photosynthetic active radiation was measured using a hand-held light quantum sensor. Daily intercepted photosynthetic active radiation (*Ipar*) and accumulated intercepted photosynthetic active radiation (*Aipar*) of the canopy were calculated using Equations 6 and 7:

(6) $$Ipar\;=\;I_{0}\;\times \;Fipar$$

(7) Aipar =∑Ipar dt

where $I_{0}$ is photosynthetic active radiation retrieved directly from the sensor, and Fipar  is the fraction of intercepted photosynthetic active radiation. In this study, *CC* derived from image segmentation in Section 2.3 was used as a proxy for Fipar, based on previous reports showing a close relationship between potato ground cover and light interception (Burstall and Harris, 1983; Haverkort et al., 1991; Mukiibi et al., 2023). Daily Fipar values between sampling dates were estimated by ‘slinear’ interpolation of the corrected *CC* data. Direct validation of this relationship under heat-stress conditions remains limited, so we adopted this equivalence as a reasonable working assumption for the current study and acknowledge the potential uncertainty associated with this approximation. For each growth stage, *Aipar* was calculated by accumulating the daily *Ipar* values over the interval extending from the date of plant emergence (4^th^ July) to the respective sampling date mentioned in Section 2.2. *RUE* was then calculated as the slope of the linear regression between cumulative dry matter and *Aipar*.

### 2.6 Statistical analysis

Linear regression analysis was performed to determine *RUE*. The statistical significance of the regression models and their respective slopes was evaluated using t-test, where *p* < 0.05 was considered statistically significant. Exact *p*-values and coefficients of determination (*R^2^*) are reported in figures to assess model fit. *R^2^* that indicate the performance of regression models were calculated using Equation 8:

(8) $$R^{2}=\;1-\frac{\sum_{i=1}^{N}{\left(P_{i}-O_{i}\right)}^{2}}{\sum_{i=1}^{N}{\left(\bar{O}-O_{i}\right)}^{2}}$$

where *N* is the number of observations, *P_i_* is the predicted value, and *O_i_* is observed value, and $\bar{O}$ expresses the mean value of measured variables.

In addition, The effects of temperature, N supply and their interactions on measured variables were analyzed using analysis of variance (ANOVA) appropriate for the split-plot experiment design. Temperature was treated as the main-plot factor, and N supply as the subplot factor when applicable. When significant treatment effects were detected, treatment means were compared using Tukey–Kramer *post hoc* tests at a significant level of *p* < 0.05. For visual clarity in figures, varying levels of statistical significance were denoted as follows: * *p* < 0.05, ** *p* < 0.01, and *** *p* < 0.001; marginal significance was indicated by *p* < 0.1. All statistical analysis and data visualization were conducted in RStudio (RStudio, PBC, Boston, MA, USA) using R.

## 3 Results

### 3.1 Effects of temperature and N supply on canopy temperature and canopy traits

**Figure 4:**
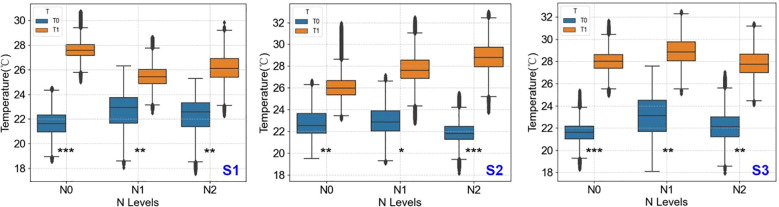
The canopy temperature distribution for different treatments at growth stages of S1, S2, and S3. Asterisks indicate significant differences between temperature regimes (T1 vs T0) within the same N treatment and stage (· *p* < 0.1, * *p* < 0.05, ** *p* < 0.01, *** *p* < 0.001).

Elevated temperature (T1) significantly increased the average, maximum, and minimum canopy temperatures compared to the control (T0) across all measured growth stages (**Figure 4**; **Table 1**). Specifically, the canopy temperature metrics under T1 increased by 2.73 °C to 7.18 °C relative to T0 across the different nitrogen levels. However, varying the N application rate did not significantly affect canopy temperature under either the T0 or T1 regime.

**Table 1:** Average, maximum and minimum canopy temperature and the difference (*D-value*) between T0 and T1 in different treatments at S1, S2, and S3 growth stages.

N treatment	Temperature	S1			S2			S3		
		T-Ave	T-Max	T-Min	T-Ave	T-Max	T-Min	T-Ave	T-Max	T-Min
N0	T0	21.57	24.23	19.31	22.72	25.89	20.02	21.61	25.11	18.73
	T1	27.64	30.04	25.17	26.07	30.27	23.40	28.00	31.53	25.59
	D-value	6.06 ***	5.81 ***	5.86 ***	3.35 **	4.38 *	3.38 ***	6.39 ***	6.42 ***	6.86 ***
N1	T0	22.76	25.33	18.78	23.08	26.69	19.26	23.31	26.17	18.95
	T1	25.49	28.38	22.95	27.70	31.68	23.80	28.85	31.61	26.13
	D-value	2.73 *	3.05 **	4.17 ***	4.63 **	4.99 ***	4.54 **	5.54 **	5.44 *	7.18 ***
N2	T0	22.30	24.83	18.06	21.89	25.01	18.56	22.16	25.83	18.65
	T1	26.10	28.97	23.50	28.83	32.11	24.46	28.03	31.14	25.16
	D-value	3.80 *	4.14 **	5.44 **	6.95 ***	7.10 ***	5.90 **	5.87 **	5.31 **	6.51 **

Asterisks indicate significant differences between temperature regimes (T1 vs T0) within the same N treatment and stage (· *p* < 0.1, * *p* < 0.05, ** *p* < 0.01, *** *p* < 0.001).

**Figure 5:**
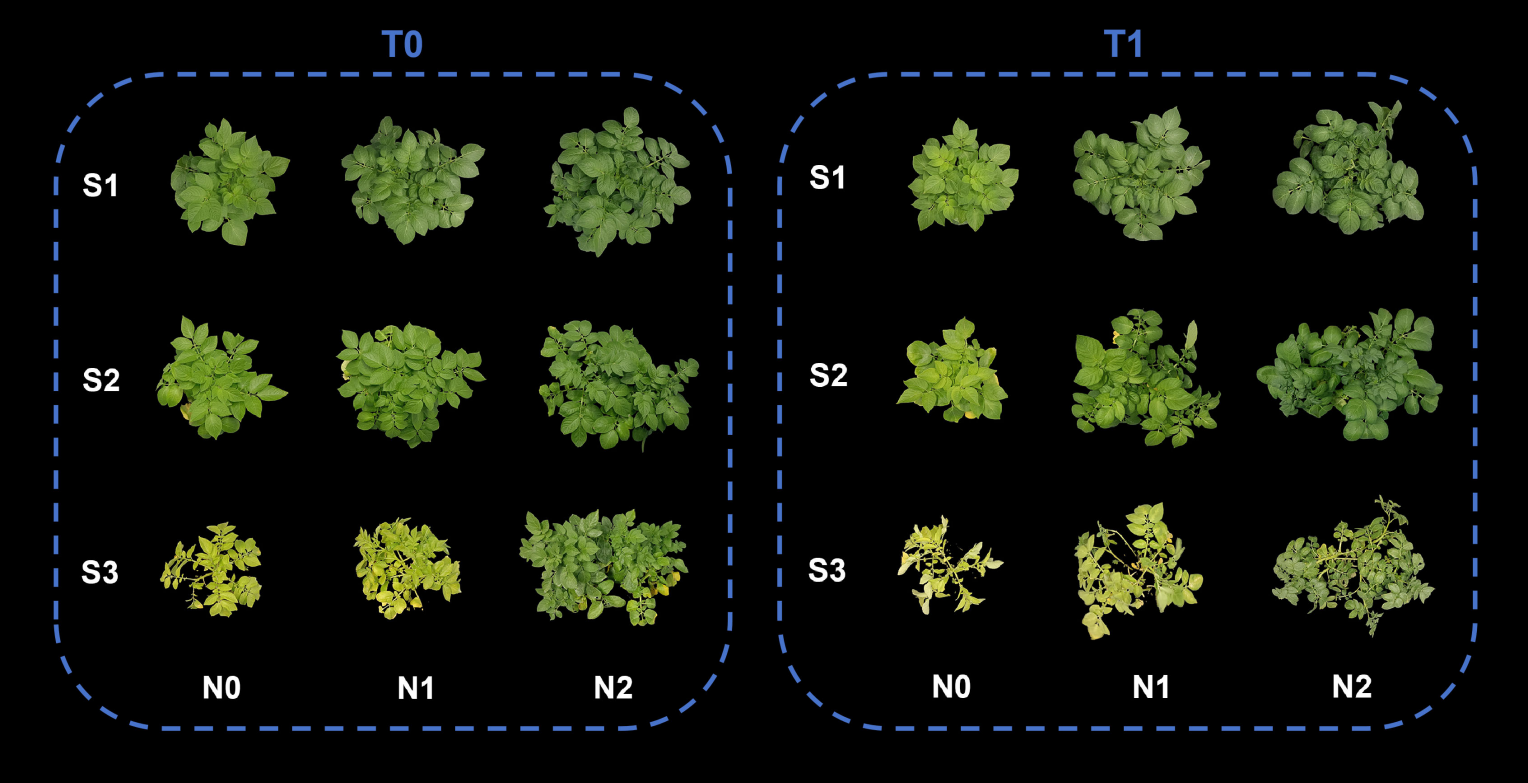
Representative canopy images of potato plants under different temperature and N treatments.

**Figure 6:**
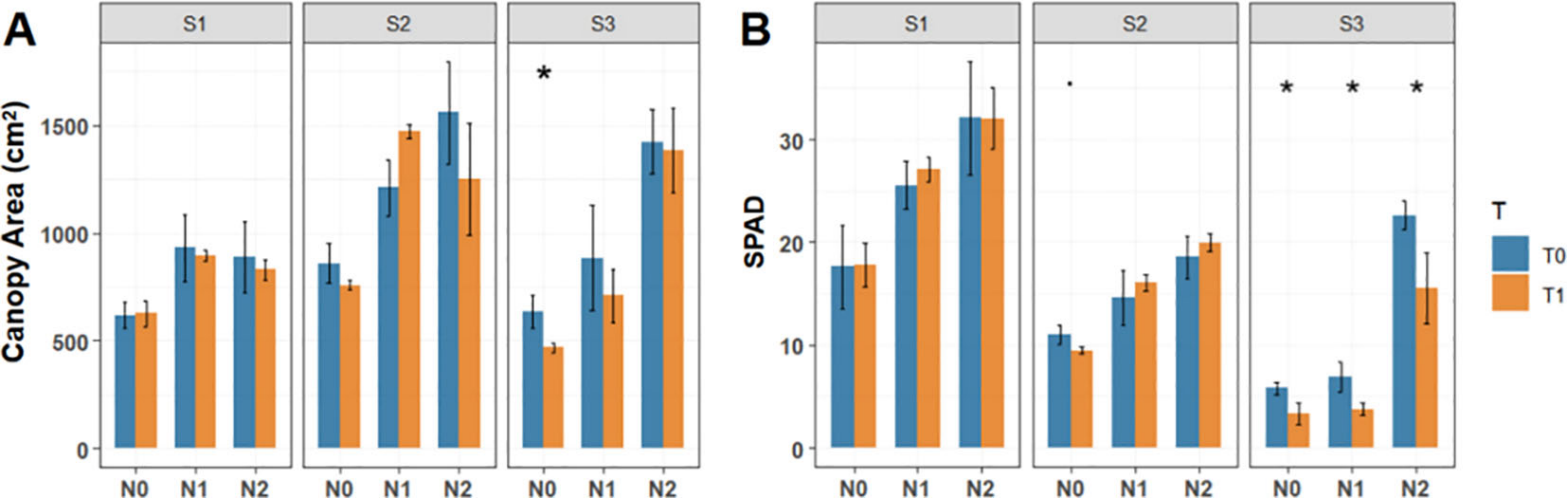
Variations of canopy area **(A)** and *SPAD* values **(B)** under different temperature and N treatments at S1, S2, and S3 growth stages. Bars represent means ± SE (n = 3). Asterisks indicate significant differences between temperature regimes (T1 vs T0) within the same N treatment and stage (· *p* < 0.1, * *p* < 0.05).

Canopy images showed visible differences among treatments (**Figure 5**), while canopy area and *SPAD* values generally increased with higher N application rates but decreased under elevated temperature (**Figure 6**). At the S3 stage, elevated temperature (T1) significantly reduced canopy area only under the nitrogen-deficient (N0) treatment (*p* < 0.05) (**Figure 6A**). The magnitude of this temperature-induced reduction in canopy area decreased from 26.31% under N0 to 2.95% under the N2 treatment. For *SPAD* values, T1 caused a significant reduction across all N treatments at the S3 stage (*p* < 0.05) (**Figure 6B**). Among these treatments, the high nitrogen supply (N2) exhibited the lowest percentage of *SPAD* reduction (31.13%) in response to high temperature. Significant T × N interactions were consistently observed at S3 (**Supplementary Table 1**).

### 3.2 N status and *C/N* affected by high temperature and N application rate

**Figure 7:**
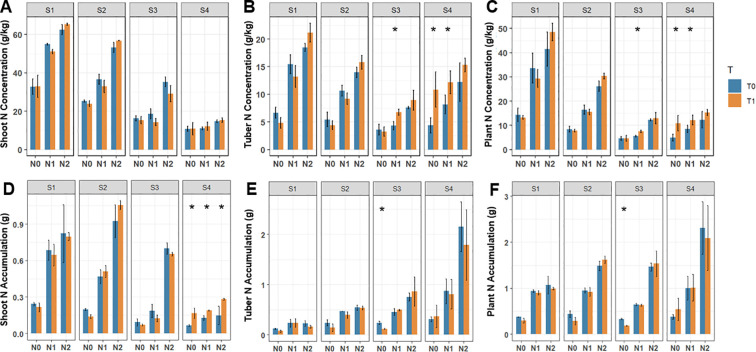
N concentration and N accumulation in different organs across developmental stages (S1, S2, S3, S4) under contrasting temperature and N supply. **(A–C)** N concentration of shoots **(A)**, tubers **(B)**, and whole plants **(C)**. **(D–F)** N accumulation in shoots **(D)**, tubers **(E)**, and whole plants **(F)**. Bars represent means ± SE (n = 3). Asterisks indicate significant differences between temperature regimes (T1 vs T0) within the same N treatment and stage (· *p* < 0.1, * *p* < 0.05, ** *p* < 0.01, *** *p* < 0.001).

N concentration and N accumulation of the shoot, tuber, and whole plant were quantified across the four growth stages (**Figure 7**).

Shoot and whole-plant N concentrations progressively declined from S1 to S4 across all treatments (**Figures 7A, C**). For tubers, N concentration initially decreased from S1 to S3, but subsequently increased at the S4 stage (**Figure 7B**). At the final S4 stage, elevated temperature (T1) increased the N concentration of both tubers and the whole plant, with the magnitude of this increase diminishing as N supply increased. Specifically, relative to T0, T1 significantly increased tuber N concentration by 148% (*p* < 0.05) and 49% (*p* < 0.05) under the N0 and N1 treatments, respectively (**Figure 7B**). Similarly, whole-plant N concentration significantly increased by 123% (*p* < 0.05) under N0 and 44% (*p* < 0.05) under N1 (**Figure 7C**). These increases may be attributed to the preferential allocation of nitrogen to tubers under heat stress, although the underlying regulatory mechanism requires further investigation. However, under the high nitrogen (N2) treatment, the observed increases in N concentration for tubers (25%) and the whole plant (25%) were not statistically significant (**Figures 7B, C**).

The patterns of N accumulation in the shoot, tuber, and whole plant further elucidated the interactive effects of temperature and N supply (**Figures 7D–F**). For the shoot, elevated temperature (T1) significantly enhanced N accumulation across all nitrogen levels at the final S4 stage. Compared to T0, the T1 treatment resulted in significant increases of 157% (*p* < 0.05), 49% (*p* < 0.05), and 90% (*p* < 0.05) in shoot N accumulation under the N0, N1, and N2 conditions, respectively (**Figure 7D**).

In contrast, the inhibitory effect of elevated temperature on tuber and whole-plant N accumulation was exclusively observed during the S3 stage under nitrogen-deficient conditions. Specifically, at S3, T1 significantly reduced tuber N accumulation by 52% (*p* < 0.05) (**Figure 7E**) and whole-plant N accumulation by 44% (*p* < 0.05) (**Figure 7F**) under the N0 treatment relative to T0. However, under the moderate (N1) and high (N2) nitrogen treatments, no statistically significant differences were detected in tuber or whole-plant N accumulation between the two temperature regimes.

**Figure 8:**
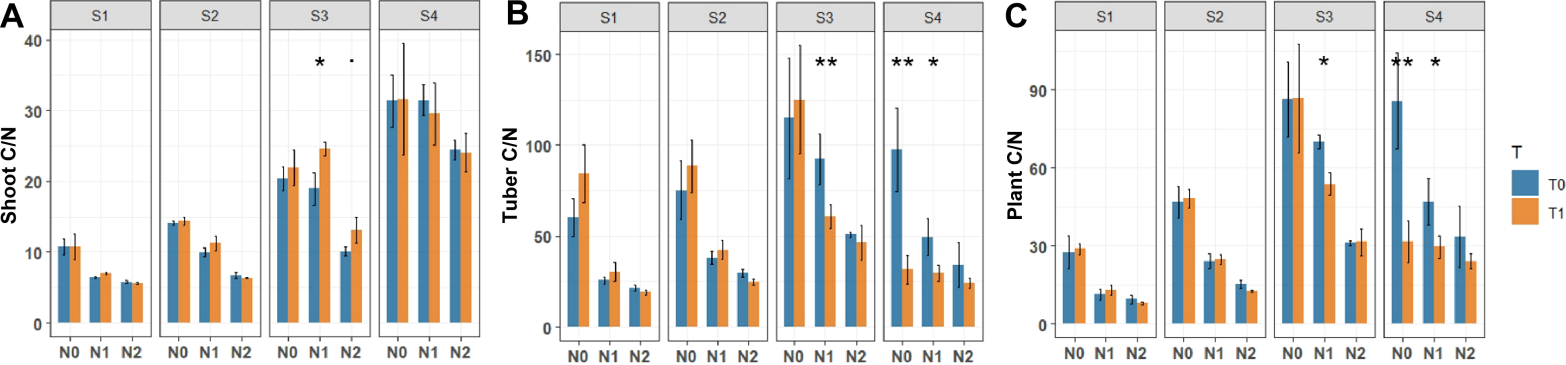
*C/N* ratio of shoot **(A)**, tuber **(B)**, and whole plant **(C)** under different temperature and nitrogen fertilizer treatments at S1, S2, S3, and S4 growth stages. Bars represent means ± SE (n = 3). Asterisks indicate significant differences between temperature regimes (T1 vs T0) within the same N treatment and stage (· *p* < 0.1, * *p* < 0.05, ** *p* < 0.01, *** *p* < 0.001).

Correspondingly, the *C/N* of the shoot, tuber, and whole plant exhibited trends inverse to those of N concentration (**Figure 8**). At the S4 stage, the magnitude of the temperature-induced reduction in the *C/N* of the tuber and whole plant depended heavily on the N application rate. Compared to T0, T1 significantly reduced the tuber *C/N* by 68% (*p* < 0.01) and 40% (*p* < 0.05) under the N0 and N1 treatments, respectively (**Figure 8B**). Concurrently, the whole-plant *C/N* was significantly reduced by 63% (*p* < 0.01) under N0 and 37% (*p* < 0.05) under N1 (**Figure 8C**). In contrast, under the N2 treatment, the reductions in the *C/N* for both the tuber (29%) and the whole plant (28%) were not statistically significant between the two temperature regimes (**Figures 8B, C**). Significant T × N interactions were consistently observed at S4 in tuber *C/N* and plant *C/N* (**Supplementary Table 1**).

### 3.3 Effects of temperature and N treatments on dry matter, biomass partitioning and tuber size distribution

#### 3.3.1 Shoot, tuber and plant dry weight

The application of nitrogen affected the dry weight of shoots, tubers, and the entire plant across all growth stages. Elevated temperature (T1) primarily affected tuber and total plant dry weights during the later developmental stages (S3 and S4).

**Figure 9:**
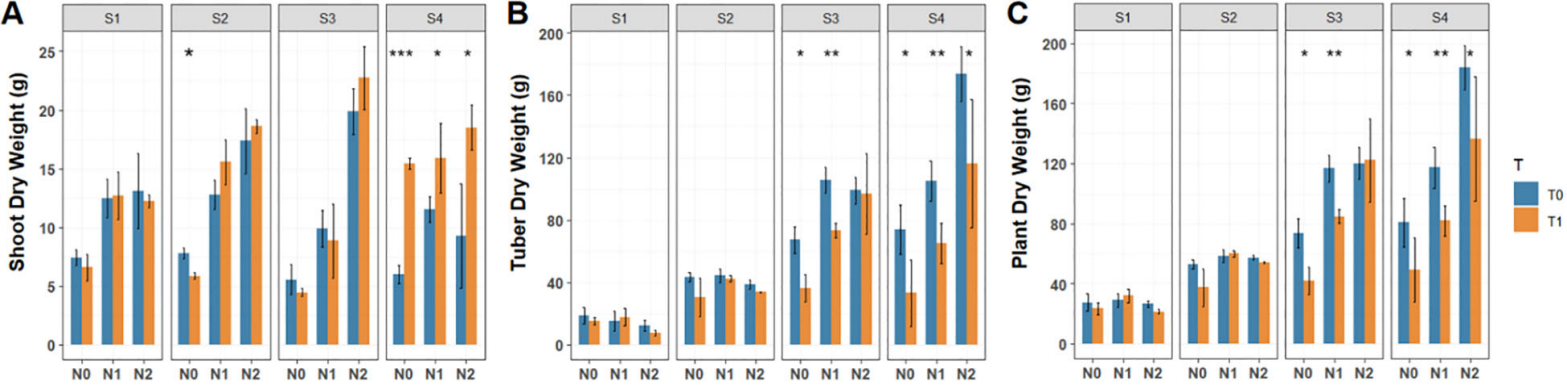
Variations in the dry weight of the shoot **(A)**, tuber **(B)**, and entire plant **(C)** under different temperature and nitrogen fertilizer treatments at S1, S2, S3, and S4 growth stages. Bars represent means ± SE (n = 3). Asterisks indicate significant differences between temperature regimes (T1 vs T0) within the same N treatment and stage (· *p* < 0.1, * *p* < 0.05, ** *p* < 0.01, *** *p* < 0.001).

For shoot dry weight, in addition to a significant reduction at S2 under N0 conditions, T1 significantly altered shoot dry weight across all N treatments at the final S4 stage. Specifically, at S4, the T1 treatment increased shoot dry weight by 156.37% (*p* < 0.001), 38.06% (*p* < 0.05), and 99.28% (*p* < 0.05) under the N0, N1, and N2 treatments, respectively, compared to T0 (**Figure 9A**).

Tuber dry weight exhibited a contrasting response to elevated temperature during the S3 and S4 stages (**Figure 9B**). At the S3 stage, T1 significantly reduced tuber dry weight under the N0 and N1 treatments (by 46.16%, *p* < 0.05; and 30.81%, *p* < 0.01, respectively). However, under the high nitrogen (N2) treatment, the difference in tuber dry weight between T0 and T1 was not statistically significant with only a 2.11% reduction. In the subsequent S4 stage, T1 significantly reduced tuber dry weight compared to T0 across all nitrogen levels. The magnitude of this reduction decreased with higher N application, from 55.13% (*p* < 0.05) under N0 to 33.26% (*p* < 0.05) under N2 (**Figure 9B**).

Similarly, the reduction in total plant dry weight under T1 was dependent on the nitrogen application rate (**Figure 9C**). At the S3 stage, T1 significantly decreased plant dry weight by 43.15% (*p* < 0.05) and 27.30% (*p* < 0.01) under the N0 and N1 treatments, respectively, whereas no significant difference was observed under the N2 treatment. At the S4 stage, plant dry weight was significantly lower under T1 across all N levels, but the percentage of reduction decreased from 38.61% under N0 to 30.15% under N1, and 26.03% under N2 (**Figure 9C**).

In addition, significant T × N interactions were consistently observed at S4 in tuber and plant dry weight but not in shoot dry weight (**Supplementary Table 1**).

#### 3.3.2 Effect of N rate and elevated T on potato biomass allocation

**Figure 10:**
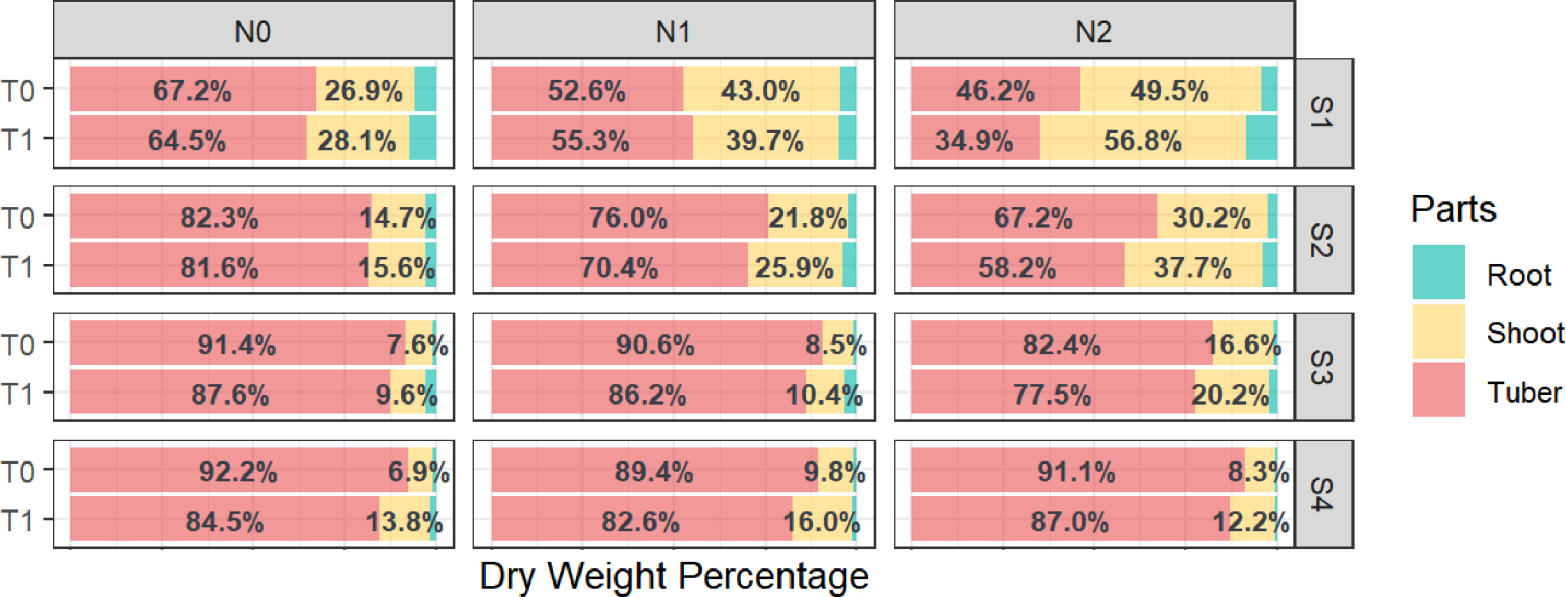
Dry weight percentage of roots, shoots, and tubers in the whole plant under different temperature and nitrogen treatments at S1, S2, S3, and S4 growth stages.

Across the four growth stages (S1–S4), distinct biomass allocation patterns were observed under different temperature and nitrogen regimes (**Figure 10**). Roots accounted for a relatively small and stable proportion (0.6%–8.3%) of total dry matter across all treatments, so subsequent analysis focused on the dynamic partitioning between shoots (source) and tubers (sink).

Under the control temperature (T0), the proportion of shoot dry weight progressively declined as plants matured, dropping from an average of 39.8% at S1 to 8.3% by S4. In contrast, high-temperature stress (T1) significantly altered this partitioning pattern. From S2 to S4, T1 consistently increased the dry matter proportion in shoots while reducing the proportion allocated to tubers relative to T0. Specifically, during the S3 and S4 phases, the tuber mass proportion under T0 ranged from 82.4% to 92.2%, whereas it was 77.5%–87.0% under T1.

The limitation of biomass translocation from shoots to tubers caused by heat stress was most evident under N0, and this limitation diminished with higher N application. For instance, at the S4 stage, the shoot dry weight proportion under T1 was 6.9% higher than under T0 for the N0 treatment, whereas this difference was 6.2% and 3.9% under N1 and N2, respectively. This shoot allocation corresponded to a reduction in the tuber proportion: under T1, the tuber fraction dropped by 7.7% (from 92.2% to 84.5%) at N0, 6.8% (from 89.4% to 82.6%) at N1, and 4.1% (from 91.1% to 87.0%) at N2 compared to T0.

#### 3.3.3 Effect of N rate and elevated T on tuber number and size distribution

**Figure 11:**
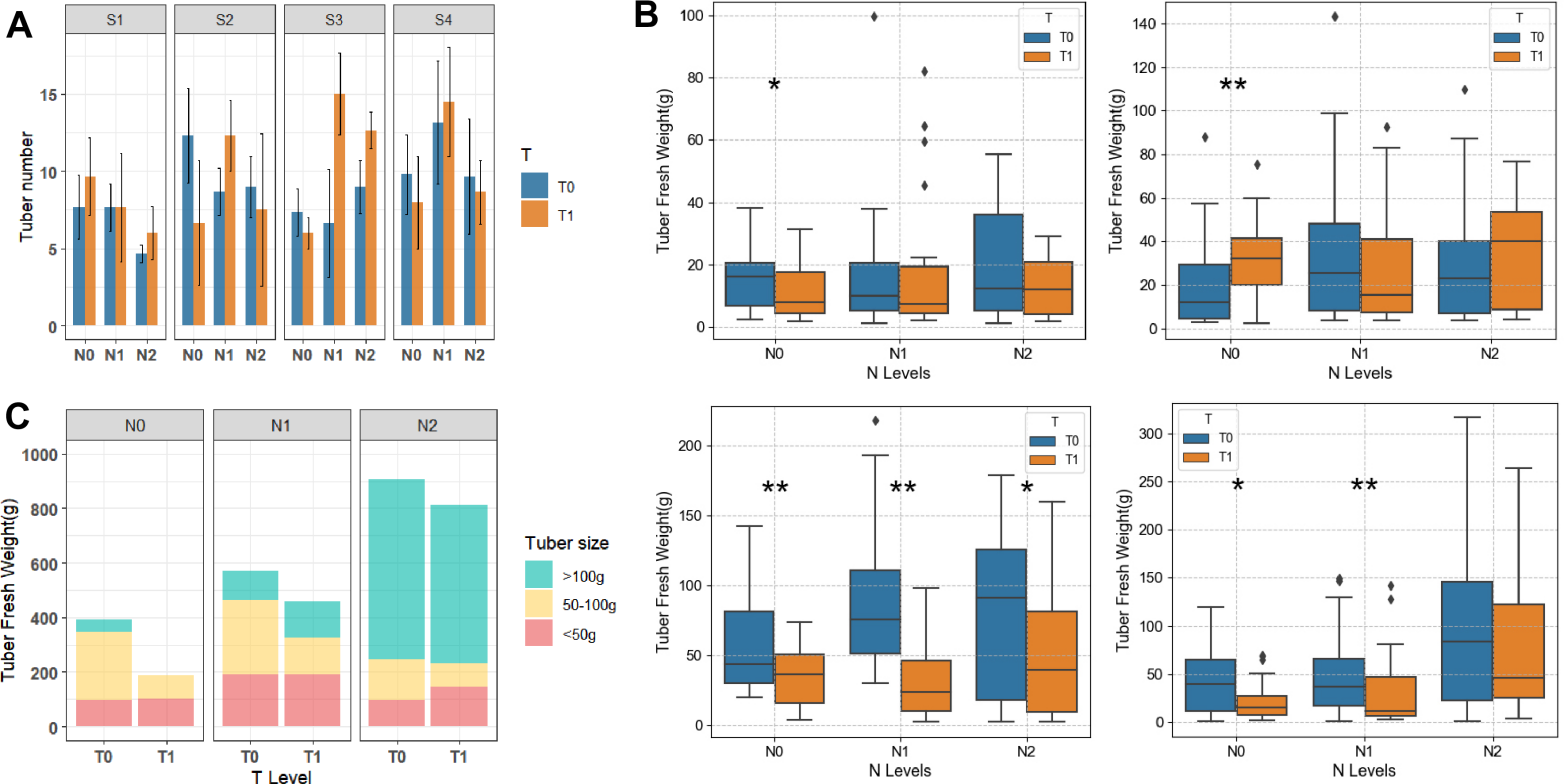
Tuber number **(A)** and the distribution of tuber fresh weight **(B)** under different temperature and nitrogen fertilizer treatments at S1, S2, S3, and S4 growth stages; the distribution of tuber fresh weight by tuber size at S4 **(C)**. Bars represent means ± SE (n = 3). Asterisks in boxplots **(B)** indicate significant differences between temperature regimes (T1 vs T0) within the same N treatment and stage (· *p* < 0.1, * *p* < 0.05, ** *p* < 0.01, *** *p* < 0.001).

Tuber number per plant was not significantly affected by either temperature or nitrogen treatments at earlier and final stages (**Figure 11A**). In contrast, the distribution of individual tuber fresh weight was significantly altered by the experimental treatments, particularly during the tuber expansion stages (S3 and S4) (**Figure 11B**). During these later stages, the median and maximum tuber fresh weights under the heat stress treatment (T1) were generally lower compared to the control temperature (T0). Consequently, the overall tuber composition shifted toward smaller tubers, with a notable reduction in the proportion of large tubers (>100g) under T1 (**Figure 11C**).

Furthermore, the magnitude of this temperature-induced reduction in tuber weight varied across different nitrogen levels. At the final S4 stage, T1 significantly decreased tuber fresh weight under the nitrogen-deficient (N0, *p* < 0.05) and moderate nitrogen (N1, *p* < 0.01) conditions relative to T0. However, under the high nitrogen application rate (N2), no statistically significant difference in tuber fresh weight was observed between the T0 and T1 regimes at S4 (**Figure 11B**).

### 3.4 Effects of temperature and N supply on radiation use efficiency

**Figure 12:**
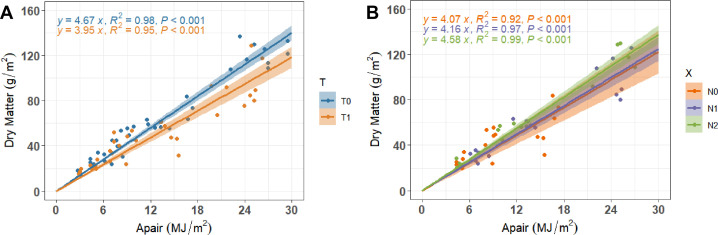
Radiation use efficiency of potato among different temperature **(A)** and nitrogen **(B)** levels.

*RUE* was quantified by performing linear regression analysis between cumulative dry matter accumulation and *Aipar*. Both T0 and T1 treatment groups exhibited strong linear correlations (*R²* > 0.95) (**Figure 12A**). High-temperature (T1) resulted in a 15.4% reduction in *RUE*, with values declining from 4.67 g/MJ under T0 to 3.95 g/MJ under T1. Furthermore, *RUE* showed a declining trend as nitrogen levels were reduced (from 4.58 g/MJ to 4.07 g/MJ), although the differences among nitrogen treatments were minimal (**Figure 12B**).

**Figure 13:**
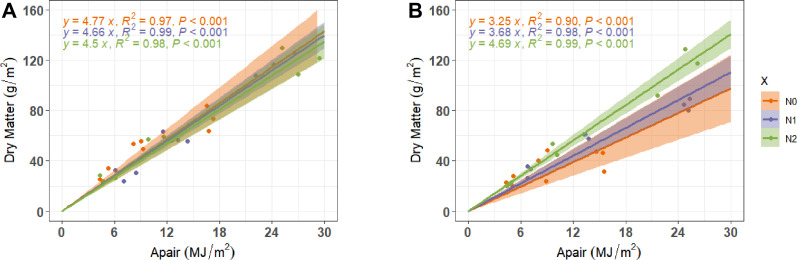
The comparison of radiation use efficiency among different N levels under T0 **(A)** and T1 **(B)**.

Under T0 condition, *RUE* values remained statistically comparable across nitrogen treatments, varying within a narrow range of 4.5 to 4.77 g/MJ, which was consistent with the observed overlap in confidence intervals (**Figure 13A**). Under T1 conditions, however, *RUE* exhibited a significant positive correlation with nitrogen application rate, rising from 3.25 (N0) to 3.68 (N1) and 4.69 (N2) g/MJ (**Figure 13B**).

**Figure 14:**
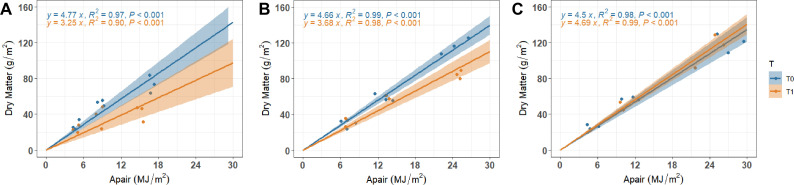
The comparison of radiation use efficiency among different temperature levels under N0 **(A)**, N1 **(B)** and N2 **(C)**.

The inhibitory impact of elevated temperature (T1) on *RUE* was highly dependent on nitrogen availability. Under nitrogen-deficient conditions (N0), heat stress exerted its most severe effect, driving a significant 31.9% reduction in *RUE* from 4.77 g/MJ (T0) to 3.25 g/MJ (T1) (**Figure 14A**). Importantly, this heat-induced penalty was progressively mitigated by increasing the nitrogen supply. Under the moderate nitrogen rate (N1), the reduction in *RUE* was narrowed to 21.0% (decreasing from 4.66 to 3.68 g/MJ) (**Figure 14B**). Remarkably, under the high nitrogen supply (N2), the detrimental effect of heat stress was effectively neutralized. The difference in *RUE* between the two temperature regimes became statistically negligible, as evidenced by the nearly overlapping regression curves and confidence intervals (**Figure 14C**).

## 4 Discussion

### 4.1 Nitrogen availability sustains canopy function and plant C-N status under non-stress conditions

Nitrogen supply strongly affected canopy development and leaf greenness, as reflected by changes in canopy area and *SPAD* (**Figure 6**). Increasing N rates expanded canopy area and increased *SPAD* values, indicating greater chlorophyll retention and delayed canopy senescence. These responses are consistent with the established role of N in sustaining leaf function and prolonging the duration of an active photosynthetic canopy (Vos and van der Putten, 1998; Peng et al., 2021). In parallel, enhanced canopy performance under adequate N coincided with greater tuber dry matter accumulation under T0 (**Figure 9B**), suggesting that N-mediated maintenance of source capacity supports carbon supply during tuber bulking.

To evaluate how N influenced biomass formation, we used the radiation-use framework in which dry matter accumulation is expressed as the product of accumulated intercepted photosynthetically active radiation (*Aipar*) and radiation use efficiency (*RUE*). Whereas *RUE* often increases with N availability in some crops such as maize and Choy sum (Kar and Kumar, 2016; He et al., 2023), potato *RUE* varied only slightly across N treatments (4.07 - 4.58 g/MJ) under control temperature (T0) (**Figure 13A**). This indicates that, within the tested N range, N primarily influenced dry matter production by modifying canopy development and thus radiation interception, while conversion efficiency at the canopy scale (*RUE*) remained relatively stable. This pattern agrees with previous observations that potato responses to N are expressed mainly through canopy development rather than large changes in unit-area photosynthetic efficiency within typical N ranges (Millard and Marshall, 1986; Bangemann et al., 2014; Zhou et al., 2016; Peng et al., 2021).

The dynamics of N concentration, N accumulation, and the *C/N* ratio provide additional context for interpreting whole-plant C-N status during development. Across stages, increasing N supply increased tissue N accumulation and was accompanied by lower *C/N* ratios in both shoots and tubers (**Figures 7**, **8**), indicating a shift in plant stoichiometry towards relatively higher N status. C-N balance is increasingly recognized as an integrative regulatory nexus linking nutrient availability with growth and metabolic regulation, mediated by central signaling networks that coordinate carbon and nitrogen metabolism (Artins et al., 2024; Fañanás-Pueyo et al., 2025). In tubers, increases in N accumulation together with decreases in *C/N* reflect a shift in sink stoichiometry during bulking and suggest enhanced N availability to support N-dependent metabolic processes associated with storage organ growth. Similar sensitivity of potato tuber development to N supply has been reported using integrated physiological and omics approaches (Ding et al., 2024). Overall, coordinated changes in shoot and tuber *C/N* highlight that N supply modulates whole-plant C-N stoichiometry and provides a physiological context for differences in dry-matter accumulation under non-stress conditions.

### 4.2 Heat stress impairs radiation use efficiency and disrupts source-sink partitioning

Elevated temperature markedly constrained potato growth during tuber development, with effects that were evident at the canopy level and propagated to whole-plant biomass accumulation and dry-matter partitioning (**Figure 4**, **6**, **9**-**11**). To interpret these responses, it is useful to separate impacts on radiation interception from impacts on conversion efficiency, using the radiation-use framework (*Aipar* × *RUE*) as an analytical tool.

At the canopy level, heat stress significantly increased canopy temperature (**Figure 4A**) and accelerated the decline in chlorophyll status, as indicated by lower *SPAD* values (**Figure 6B**), consistent with reduced canopy functional performance under elevated temperature. In contrast, canopy area was significantly reduced by heat only under the N0 treatment at S3 (**Figure 6A**). Under adequate N supply, canopy area remained relatively stable, although a slight decreasing trend under heat stress was observed, suggesting that potential canopy light interception was only marginally affected. Despite the relatively stable canopy area, *RUE* declined substantially under heat stress, indicating that the conversion of intercepted radiation into biomass became less efficient. Heat-induced reductions in photosynthetic performance and photochemical efficiency have been widely reported and are often associated with constraints on photosynthetic processes and increased oxidative pressure on the photosynthetic apparatus (Chauhan et al., 2023; Zahra et al., 2023). These established responses provide a physiological context for the decline in *RUE* observed here.

The reduction in *RUE* was accompanied by lower total dry matter accumulation, with tuber biomass more strongly affected than aboveground biomass (**Figure 9**). Heat stress also shifted dry-matter partitioning away from tubers by increasing the fraction of biomass retained in shoots during the critical bulking stages (S3-S4) (**Figures 10**, **11**). This shift highlights a partial decoupling between canopy carbon supply and tuber carbon utilization: limitations in tuber filling can become a dominant constraint on yield formation even when canopy structure is only modestly affected (Lehretz et al., 2020). Recent potato studies support this interpretation, reporting that yield loss under elevated temperature can arise primarily from impaired tuber filling rather than reduced tuber initiation (Koch et al., 2024; Guillemette et al., 2025). Consistent with this interpretation, heat-related reductions in tuber biomass were most pronounced during S3–S4, indicating that elevated temperature primarily constrained dry-matter allocation to tubers during bulking, whereas tuber number was not significantly affected at earlier stages (**Figure 11A**).

Collectively, elevated temperature constrains potato productivity through a multi-level mechanism: it first alters canopy physiological status, then impairs photosynthetic efficiency and *RUE*, leading to reduced biomass production, and ultimately disrupts dry-matter partitioning to tubers, indicating impaired sink utilization during bulking. These interconnected processes converge to limit tuber formation and yield potential. Consequently, this raises the question of whether optimized nitrogen application strategies can mitigate the negative impacts of high temperature.

### 4.3 Nitrogen supply mitigates heat-induced physiological impairments by sustaining canopy function and dry matter partitioning to tubers

The interaction between temperature and N supply was most evident during tuber bulking (S3-S4), when canopy function, conversion efficiency, and dry-matter allocation jointly determined the magnitude of yield loss under heat (**Figure 6**, **10**, **11**, **13**, **14**). Across these stages, increasing N supply moderated heat-associated declines in canopy function and *RUE* and reduced the heat-induced restriction on dry-matter partitioning from shoots to tubers.

Under heat stress, canopy traits and conversion efficiency became strongly dependent on N supply. Canopy development and greenness (canopy area and *SPAD*) were comparatively stable under high N (N2), whereas low-N plants (N0) exhibited a stronger heat-associated decline in canopy greenness and green canopy area (**Figure 6**). Given that heat stress can accelerate chlorophyll loss and affect turnover of photosynthetic proteins, including components of the *PSII* repair cycle (e.g., D1 turnover) (Zahra et al., 2023; Didaran et al., 2024), the N-associated maintenance of canopy greenness observed here is consistent with improved preservation of photosynthetic capacity under stress. Moreover, N deficiency is known to promote degradation of Rubisco and photosystem proteins and to accelerate senescence-related proteolysis (Sakuraba, 2022), providing additional context for the stronger canopy deterioration observed under low N.

In parallel, *RUE* showed little variation among N treatments under T0 (**Figure 13A**), but under T1 it declined sharply at low N and increased progressively with N rate (**Figure 13B**). Accordingly, the *RUE* difference between T0 and T1 narrowed with increasing N supply and became statistically non-significant at N2 (**Figure 14**). Because heat stress can constrain photosynthesis through processes that include impaired photochemistry and limitations on Rubisco activation (Zahra et al., 2023), and Rubisco activase is a known determinant of maintaining Rubisco activation at elevated temperature (Qu et al., 2021; Salesse-Smith et al., 2025), the observed N-dependence of *RUE* under heat is consistent with N supporting canopy-scale conversion efficiency under stress. These responses provide a physiological context for the N-dependent variation in *RUE* observed here, although the underlying biochemical mechanisms were not directly examined in this study. Although the absolute magnitude of *RUE* depends on the method used to approximate intercepted radiation, the consistent responses of *SPAD*, canopy area, and biomass accumulation support the robustness of the treatment comparisons.

Heat stress altered dry-matter partitioning by increasing the fraction of biomass retained in shoots during late development, but this shift was progressively attenuated as N supply increased. At S4, the shoot fraction under T1 exceeded that under T0 by 6.9%, 6.2%, and 3.9% under N0, N1, and N2, respectively (**Figure 10**), indicating that higher N supply partially alleviated the heat-induced restriction on dry-matter allocation from shoots to tubers. Consistent with a filling limitation rather than impaired initiation, tuber number showed no significant change at earlier stages (**Figure 11A**), whereas heat-related reductions in tuber biomass were most pronounced during S3-S4 (**Figure 9B**). At harvest, the reduction in tuber dry weight under heat declined from 55% at N0 to 33% at N2 (**Figure 9B**), demonstrating that adequate N reduced the magnitude of heat-induced yield loss by sustaining tuber filling during bulking.

In addition to allocation changes, N supply also narrowed temperature-related differences in whole-plant and tuber *C/N* under T1 (**Figure 8**), indicating tighter coordination between carbon gain and N status during bulking. While *C/N* does not directly quantify transport rates or enzyme activities, a lower and more stable tuber *C/N* is consistent with improved N status of the sink tissue, which may support N-dependent metabolic capacity associated with storage organ growth and starch accumulation. According to the recently proposed *C/N* balance signal theory (Fañanás-Pueyo et al., 2025), plants perceive this internal *C/N* as a crucial systemic signal to regulate developmental phase transitions and source-sink relationships. An optimal N status helps maintain an appropriate *C/N* balance, which prevents the excessive retention of assimilates in the vegetative organs and instead triggers the efficient export of photosynthates from the shoot to the tuber. At the molecular level, this source-sink coordination is driven by complementary signaling networks. As demonstrated by recent studies (Ding et al., 2024), N availability modulates C-N related regulation at the sink level by directly regulating the expression profiles of key sucrose transporters (e.g., the SUT and SWEET gene families) and acting in synergy with plant hormone signals, such as abscisic acid (ABA) and auxin. Collectively, these allocation and stoichiometric responses suggest that N supply supports more effective source-sink coordination under heat, thereby limiting the extent to which elevated temperature constrains tuber filling.

Together, these results indicate that adequate N supply mitigates heat-induced tuber yield loss mainly by sustaining canopy function and *RUE* and by alleviating the restriction of dry-matter partitioning to tubers during bulking. This provides a physiological basis for understanding how N status modifies potato heat tolerance. However, it should be noted that while this study provides mechanistic insights into the interactive effects of temperature and nitrogen under highly controlled climate chamber conditions, there are inherent limitations. First, although rigorous environmental control significantly minimized background noise and confounding factors to clearly isolate physiological responses, the trial was conducted over a single experimental cycle. Translating these findings to open-field agriculture requires caution, as pot experiments constrain root growth space and may differ from field conditions regarding soil nutrient uniformity. Furthermore, this study utilized a single cultivar (*Zhongshu 5*). Future multi-year field trials incorporating multiple potato varieties with varying degrees of heat tolerance are necessary to verify the universality of these physiological interactions under complex, fluctuating natural environments.

## 5 Conclusions

Elevated temperature reduced potato tuber yield mainly by impairing radiation use efficiency and restricting dry matter allocation to tubers during bulking. Within the nitrogen application range tested in this study (0 to 150 kg ha^-^¹), adequate N supply alleviated these heat-induced constraints by sustaining canopy function, stabilizing *RUE*, and reducing biomass retention in shoots, thereby improving dry-matter partitioning to tubers under thermal stress. These findings provide a physiological basis for optimizing N management to reduce heat-induced yield loss in potato.

## Data Availability

The raw data supporting the conclusions of this article will be made available by the authors, without undue reservation.

## References

[B1] Artins A. Martins M. C. M. Meyer C. Fernie A. R. Caldana C. (2024). Sensing and regulation of C and N metabolism - novel features and mechanisms of the TOR and SnRK1 signaling pathways. Plant J. 118, 1268–1280. doi: 10.1111/tpj.16684 38349940

[B2] Bangemann L.-W. Sieling K. Kage H. (2014). The effect of nitrogen and late blight on crop growth, solar radiation interception and yield of two potato cultivars. Field Crops Res. 155, 56–66. doi: 10.1016/j.fcr.2013.09.022 38826717

[B3] Burstall L. Harris P. M. (1983). The estimation of percentage light interception from leaf area index and percentage ground cover in potatoes. J. Agric. Sci. 100, 241–244. doi: 10.1017/S0021859600032676 41292463

[B4] Chauhan J. Prathibha M. D. Singh P. Choyal P. Mishra U. N. Saha D. . (2023). Plant photosynthesis under abiotic stresses: Damages, adaptive, and signaling mechanisms. Plant Stress 10. doi: 10.1016/j.stress.2023.100296 38826717

[B5] Dahal K. Li X.-Q. Tai H. Creelman A. Bizimungu B. (2019). Improving potato stress tolerance and tuber yield under a climate change scenario - a current overview. Front. Plant Sci. 10. doi: 10.3389/fpls.2019.00563 31139199 PMC6527881

[B6] Didaran F. Kordrostami M. Ghasemi-Soloklui A. A. Pashkovskiy P. Kreslavski V. Kuznetsov V. . (2024). The mechanisms of photoinhibition and repair in plants under high light conditions and interplay with abiotic stressors. J. Photochem. Photobiol. B. Biol. 259, 113004. doi: 10.1016/j.jphotobiol.2024.113004 39137703

[B7] Ding K. Shan Y. Wang L. Zhang Y. Tian G. (2024). Transcriptomics combined with physiological analysis and metabolomics revealed the response of potato tuber formation to nitrogen. BMC Plant Biol. 24, 1109. doi: 10.1186/s12870-024-05758-2 39573986 PMC11583798

[B8] Fañanás-Pueyo I. Carrera-Castaño G. Pernas M. Oñate-Sánchez L. (2025). Signalling and regulation of plant development by carbon/nitrogen balance. Physiol. Plant 177, e70228. doi: 10.1111/ppl.70228 40269445 PMC12018728

[B9] Fang X. Xiang Z. Ma H. Wang F. Wang Q. Li P. . (2023). Effect of N fertilizer dosage and base/topdressing ratio on potato growth characteristics and yield. Agronomy 13, 909. doi: 10.3390/agronomy13030909 30654563

[B10] Fleisher D. H. Timlin D. J. Reddy V. R. (2006). Temperature influence on potato leaf and branch distribution and on canopy photosynthetic rate. Agron. J. 98, 1442–1452. doi: 10.2134/agronj2005.0322

[B11] Guillemette A. M. Hernández Casanova G. Hamilton J. P. Pokorná E. Dobrev P. I. Motyka V. . (2025). The physiological and molecular responses of potato tuberization to projected future elevated temperatures. Plant Physiol. 197, kiae664. doi: 10.1093/plphys/kiae664 39688842 PMC11683837

[B12] Gururani M. A. Venkatesh J. Tran L. S. (2015). Regulation of photosynthesis during abiotic stress-induced photoinhibition. Mol. Plant 8, 1304–1320. doi: 10.1016/j.molp.2015.05.005 25997389

[B13] Hancock R. D. Morris W. L. Ducreux L. J. Morris J. A. Usman M. Verrall S. R. . (2014). Physiological, biochemical and molecular responses of the potato (Solanum tuberosum L.) plant to moderately elevated temperature. Plant Cell Environ. 37, 439–450. doi: 10.1111/pce.12168 23889235

[B14] Haverkort A. J. Uenk D. Veroude H. Van De Waart M. (1991). Relationships between ground cover, intercepted solar radiation, leaf area index and infrared reflectance of potato crops. Potato Res. 34, 113–121. doi: 10.1007/BF02358105 30311153

[B15] He Y. Wang Z. Sun S. Zhu L. Li Y. Wang X. . (2023). Using crop intercepted solar radiation and vegetation index to estimate dry matter yield of Choy Sum. Front. Plant Sci. 14, 1208404. doi: 10.3389/fpls.2023.1208404 37790780 PMC10544932

[B16] Hijmans R. J. (2003). The effect of climate change on global potato production. Am. J. Potato Res. 80, 271–279. doi: 10.1007/BF02855363 30311153

[B17] Jongschaap R. E. E. Booij R. (2004). Spectral measurements at different spatial scales in potato: relating leaf, plant and canopy nitrogen status. Int. J. Appl. Earth Obs. Geoinf. 5, 205–218. doi: 10.1016/j.jag.2004.03.002 38826717

[B18] Kar G. Kumar A. (2016). Radiation utilization efficiency and surface energy exchange of winter maize (Zea mays L.) under different irrigation regimes. J. Agrometeorol. 18, 190–195. doi: 10.54386/jam.v18i2.934

[B19] Koch L. Lehretz G. G. Sonnewald U. Sonnewald S. (2024). Yield reduction caused by elevated temperatures and high nitrogen fertilization is mitigated by SP6A overexpression in potato (Solanum tuberosum L.). Plant J. 117, 1702–1715. doi: 10.1111/tpj.16679 38334712

[B20] Lehretz G. G. Sonnewald S. Lugassi N. Granot D. Sonnewald U. (2020). Future-proofing potato for drought and heat tolerance by overexpression of hexokinase and SP6A. Front. Plant Sci. 11, 614534. doi: 10.3389/fpls.2020.614534 33510758 PMC7835534

[B21] Levy D. Veilleux R. E. (2007). Adaptation of potato to high temperatures and salinity-a review. Am. J. Potato Res. 84, 487–506. doi: 10.1007/BF02987885 30311153

[B22] Liu K. Deng J. Lu J. Wang X. Lu B. Tian X. . (2019). High nitrogen levels alleviate yield loss of super hybrid rice caused by high temperatures during the flowering stage. Front. Plant Sci. 10, 357. doi: 10.3389/fpls.2019.00357 30972091 PMC6443885

[B23] Liu K. Du J. Zhong Y. Shen Z. Yu X. (2021). The response of potato tuber yield, nitrogen uptake, soil nitrate nitrogen to different nitrogen rates in red soil. Sci. Rep. 11, 22506. doi: 10.1038/s41598-021-02086-5 34795355 PMC8602656

[B24] Liu L. Hu C. Olesen J. E. Ju Z. Yang P. Zhang Y. (2013). Warming and nitrogen fertilization effects on winter wheat yields in northern China varied between four years. Field Crops Res. 151, 56–64. doi: 10.1016/j.fcr.2013.07.006 38826717

[B25] Mathur S. Agrawal D. Jajoo A. (2014). Photosynthesis: response to high temperature stress. J. Photochem. Photobiol. B. Biol. 137, 116–126. doi: 10.1016/j.jphotobiol.2014.01.010 24796250

[B26] Millard P. Marshall B. (1986). Growth, nitrogen uptake and partitioning within the potato (Solatium tuberosum L.) crop, in relation to nitrogen application. J. Agric. Sci. 107, 421–429. doi: 10.1017/S0021859600087220 41292463

[B27] Monteith J. L. (1977). Climate and the efficiency of crop production in Britain. Philos. Trans. R. Soc. London. B. Biol. Sci. 281, 277–294. doi: 10.1098/rstb.1977.0140 34341189

[B28] Mukiibi A. Franke A. C. Steyn J. M. (2023). Determination of crop coefficients and evapotranspiration of potato in a semi-arid climate using canopy state variables and satellite-based NDVI. Remote Sens. 15, 4579. doi: 10.3390/rs15184579 30654563

[B29] Parent B. Tardieu F. (2012). Temperature responses of developmental processes have not been affected by breeding in different ecological areas for 17 crop species. New Phytol. 194, 760–774. doi: 10.1111/j.1469-8137.2012.04086.x 22390357

[B30] Paul S. Das M. K. Baishya P. Ramteke A. Farooq M. Baroowa B. . (2017). Effect of high temperature on yield associated parameters and vascular bundle development in five potato cultivars. Sci. Hortic. 225, 134–140. doi: 10.1016/j.scienta.2017.06.061 38826717

[B31] Peng J. Manevski K. Kørup K. Larsen R. Andersen M. N. (2021). Random forest regression results in accurate assessment of potato nitrogen status based on multispectral data from different platforms and the critical concentration approach. Field Crops Res. 268, 108158. doi: 10.1016/j.fcr.2021.108158 38826717

[B32] Qu Y. Sakoda K. Fukayama H. Kondo E. Suzuki Y. Makino A. . (2021). Overexpression of both Rubisco and Rubisco activase rescues rice photosynthesis and biomass under heat stress. Plant Cell Environ. 44, 2308–2320. doi: 10.1111/pce.14051 33745135

[B33] Rykaczewska K. (2013). The impact of high temperature during growing season on potato cultivars with different response to environmental stresses. Am. J. Plant Sci. 4, 2386–2393. doi: 10.4236/ajps.2013.412295

[B34] Sakuraba Y. (2022). Molecular basis of nitrogen starvation-induced leaf senescence. Front. Plant Sci. 13-2022. doi: 10.3389/fpls.2022.1013304 36212285 PMC9538721

[B35] Salesse-Smith C. E. Wang Y. Long S. P. (2025). Increasing Rubisco as a simple means to enhance photosynthesis and productivity now without lowering nitrogen use efficiency. New Phytol. 245, 951–965. doi: 10.1111/nph.20298 39688507 PMC11711929

[B36] Saravia D. Farfán-Vignolo E. R. Gutiérrez R. De Mendiburu F. Schafleitner R. Bonierbale M. . (2016). Yield and physiological response of potatoes indicate different strategies to cope with drought stress and nitrogen fertilization. Am. J. Potato Res. 93, 288–295. doi: 10.1007/s12230-016-9505-9 30311153

[B37] Sinclair T. R. Muchow R. C. (1999). Radiation use efficiency. Adv. Agron. 65, 215–265. doi: 10.1016/S0065-2113(08)60914-1

[B38] Singh B. Kukreja S. Goutam U. (2020). Impact of heat stress on potato (Solanum tuberosum L.): present scenario and future opportunities. J. Hortic. Sci. Biotechnol. 95, 407–424. doi: 10.1080/14620316.2019.1700173 37339054

[B39] Struik P. C. Geertsema J. Custers C. H. M. G. (1989). Effects of shoot, root and stolon temperature on the development of the potato (Solanum tuberosum L.) plant. I. Development of the haulm. Potato Res. 32, 133–141. doi: 10.1007/BF02358225 30311153

[B40] Thornton P. Ericksen P. Herrero M. Challinor A. (2014). Climate variability and vulnerability to climate change: a review. Global Change Biol. 20, 3313–3328. doi: 10.1111/gcb.12581 24668802 PMC4258067

[B41] Vos J. van der Putten P. E. L. (1998). Effect of nitrogen supply on leaf growth, leaf nitrogen economy and photosynthetic capacity in potato. Field Crops Res. 59, 63–72. doi: 10.1016/S0378-4290(98)00107-5

[B42] Wang C. Zang H. Liu J. Shi X. Li S. Chen F. . (2020). Optimum nitrogen rate to maintain sustainable potato production and improve nitrogen use efficiency at a regional scale in China. A meta-analysis. Agron. Sustain. Dev. 40, 37. doi: 10.1007/s13593-020-00640-5 30311153

[B43] Waraich E. A. Ahmad R. Halim A. Aziz T. (2012). Alleviation of temperature stress by nutrient management in crop plants: a review. J. Soil Sci. Plant Nutr. 12, 221–244. doi: 10.4067/S0718-95162012000200003

[B44] Wolf S. Marani A. Rudich J. (1991). Effect of temperature on carbohydrate metabolism in potato plants. J. Exp. Bot. 42, 619–625. doi: 10.1093/jxb/42.5.619 12432039

[B45] Zahra N. Hafeez M. B. Ghaffar A. Kausar A. Zeidi M. A. Siddique K. H. M. . (2023). Plant photosynthesis under heat stress: Effects and management. Environ. Exp. Bot. 206, 105178. doi: 10.1016/j.envexpbot.2022.105178 38826717

[B46] Zhang S. Ye H. Kong L. Li X. Chen Y. Wang S. . (2024). Multivariate analysis compares and evaluates heat tolerance of potato germplasm. Plants 13, 142–142. doi: 10.3390/plants13010142 38202450 PMC10781149

[B47] Zhou Z. Andersen M. N. Plauborg F. (2016). Radiation interception and radiation use efficiency of potato affected by different N fertigation and irrigation regimes. Eur. J. Agron. 81, 129–137. doi: 10.1016/j.eja.2016.09.007 38826717

[B48] Zhou Z. Plauborg F. Kristensen K. Andersen M. N. (2017). Dry matter production, radiation interception and radiation use efficiency of potato in response to temperature and nitrogen application regimes. Agric. For. Meteorol. 232, 595–605. doi: 10.1016/j.agrformet.2016.10.017 38826717

