# Supplementary material for: Nitrogen supply mitigates heat-driven potato yield loss by sustaining radiation use efficiency and dry matter partitioning to tubers
Source: Front Plant Sci. 2026 Jun 10;17:1846993. doi: 10.3389/fpls.2026.1846993 (PMC13290516; doi:10.3389/fpls.2026.1846993)
Supplement: Supplementary file 1 [file Table1.docx]

**Supplementary Table S1.** Summary of statistical significance (*p*-values) from one-way and two-way analysis of variances (ANOVA) for the main effects of temperature (T) and nitrogen (N) and their interactions (T×N). Asterisks indicate significant differences : · *p* < 0.1, * *p* < 0.05, ** *p* < 0.01, *** *p* < 0.001; ns: not significant (*p* ≥ 0.1); "--": not determined (no data available). *C/N*: Carbon/Nitrogen ratio.

|  |  | **S1** | **S2** | **S3** | **S4** |
| --- | --- | --- | --- | --- | --- |
| **Canopy Area** | T | ns | ns | ns | -- |
|  | N | *** | *** | *** | -- |
|  | T×N | ns | ns | ns | -- |
| **SPAD** | T | ns | ns | *** | -- |
|  | N | *** | *** | *** | -- |
|  | T×N | ns | . | ** | -- |
| **Shoot N Concentration** | T | ns | ns | ** | ns |
|  | N | *** | *** | *** | *** |
|  | T×N | ns | . | ns | * |
| **Tuber N Concentration** | T | ns | ns | ** | *** |
|  | N | *** | *** | *** | *** |
|  | T×N | ns | . | . | ns |
| **Plant N Concentration** | T | ns | ns | ** | *** |
|  | N | *** | *** | *** | *** |
|  | T×N | ns | . | . | ns |
| **Shoot N Accumulation** | T | ns | ns | ** | *** |
|  | N | *** | *** | *** | *** |
|  | T×N | ns | ns | ns | ns |
| **Tuber N Accumulation** | T | ns | ** | ns | ns |
|  | N | *** | *** | *** | *** |
|  | T×N | ns | ns | ns | ns |
| **Plant N Accumulation** | T | ns | ns | ns | ns |
|  | N | *** | *** | *** | *** |
|  | T×N | ns | * | ns | ns |
| **Shoot *C/N*** | T | ns | ns | ** | ns |
|  | N | *** | *** | *** | *** |
|  | T×N | ns | ns | ns | ns |
| **Tuber *C/N*** | T | * | ns | ns | *** |
|  | N | *** | *** | *** | *** |
|  | T×N | ns | ns | ns | ** |
| **Plant *C/N*** | T | ns | ns | ns | *** |
|  | N | *** | *** | *** | *** |
|  | T×N | ns | * | ns | ** |
| **Shoot Dry Matter** | T | ns | ns | ns | *** |
|  | N | *** | *** | *** | ** |
|  | T×N | ns | . | ns | ns |
| **Tuber Dry Matter** | T | ns | ns | *** | *** |
|  | N | ** | ** | *** | *** |
|  | T×N | ns | ns | * | * |
| **Plant Dry Matter** | T | ns | ns | *** | *** |
|  | N | *** | *** | *** | *** |
|  | T×N | ns | * | * | * |
